# Trends in Aortic Stenosis Mortality Among Older Adults in the United States from 1999 to 2020

**DOI:** 10.3390/jcm14238276

**Published:** 2025-11-21

**Authors:** Muhammad Ahmad, Salman Zahid, Mustafa Shehzad, Dawood Shehzad, Evan Shalen, Hind Rahmouni, Muhammad Raza, Craig Basman, Marian Vandyck-Acquah, Ryan Kaple

**Affiliations:** 1 Department of Medicine, Khyber Medical College, Peshawar 25120, Pakistan; muhammadahmad.mail44@gmail.com; 2 Department of Cardiovascular Medicine, Knight Cardiovascular Institute, Oregon Health and Science University, Portland, OR 97239, USA; zahidsa@ohsu.edu (S.Z.);; 3 Department of Internal Medicine, Hackensack University Medical Center, Hackensack, NJ 07601, USA; mustafa.shehzad@hmhn.org; 4 Department of Internal Medicine, Sanford School of Medicine, University of South Dakota, Sioux Falls, SD 57107, USA; daud.shehzad@usd.edu; 5 Department of Cardiology, Deborah Heart and Lung Center, Brown Mills, NJ 08015, USA; 6 Department of Cardiology, Hackensack Meridian School of Medicine, Hackensack University Medical Center, Hackensack, NJ 07601, USA; craig.basman@hmhn.org (C.B.);

**Keywords:** aortic stenosis, cardiovascular, mortality, CDC WONDER, USA

## Abstract

**Background:** Aortic stenosis (AS) represents a prevalent valvular condition in older adults, associated with significant morbidity and mortality. The objective of the study was to examine trends in mortality related to AS in the United States (U.S.). **Methods:** The U.S. CDC WONDER dataset was analyzed, extracting age-adjusted mortality rates (AAMR) per 100,000 and calculating annual percentage change (APC) through Joinpoint regression. The results were stratified to identify temporal, sex-specific, racial/ethnic, and regional differences. **Results:** From 1999 to 2020, 267,515 deaths among older adults (>65 years old) were attributed to AS, with the AAMR declining from 28.00 to 23.69. Males had a higher AAMR (30.35) compared to females (27.42), though more deaths occurred in females (164,104 vs. 103,411). Non-Hispanic (NH) Whites exhibited the greatest AAMR (31.61), trailed by NH American Indian/Alaska Native individuals (16.62), whereas NH Asians/Pacific Islanders had the least (11.50). Significant state-wise variations were noted, with AAMRs ranging from 60.55 in Oregon to 17.23 in Mississippi, and 19 states depicting a concerning rise over the study duration. Regionally, the Northeast (32.09) had the highest AAMRs, while the South (23.06) had the lowest. Micropolitan (32.28) and noncore (28.43) areas reported higher AAMRs compared to large central metropolitan areas (24.32). **Conclusions:** While there is a trend towards decreased mortality due to AS in the U.S., significant disparities based on race, sex, and region persist and may be worsening. The underlying causes of these discrepancies require further investigation, and targeted strategies must be developed to address them effectively.

## 1. Introduction

Aortic stenosis (AS) represents the most prevalent valvular disease, affecting 2–5% adults above 65 years of age. Studies in those aged 75 and older have reported that approximately 12.4% are affected by AS, with severe disease occurring in 3.4% [1]. The prevalence of AS shows a clear age-related increase, affecting about 0.2% of individuals in their 50s, roughly 1% in their 60s, nearly 4% in their 70s, and approaching 10% in those in their 80s [2].

AS can be caused by congenital, degenerative, and rheumatic processes, leading to progressive valvular fibrosis and calcification. This leads to a gradual obstruction of the aortic orifice, resulting in left ventricular pressure overload and the eventual onset of symptoms. Without aortic valve replacement (AVR), the prognosis is fatal within 2 to 3 years [3].

In recent decades, transcatheter aortic valve replacement (TAVR) has shown promise for patients who are poor candidates for surgical valve replacement [4]. The number of patients receiving surgical or transcatheter treatment is rising annually, with improving outcomes.

However, the temporal, sex-based, racial, and geographic variations of AS-related mortality trends in the U.S. remain uncertain. We hypothesize that outcomes have improved over time with advancements in management, leading to lower mortality rates.

## 2. Methodology

This study drew upon publicly available data from the Centers for Disease Control and Prevention’s Wide-Ranging Online Data for Epidemiologic Research (CDC WONDER) database, which compiles mortality information derived from death certificates across all fifty states and the District of Columbia.

## 3. Study Setting and Population

We carried out a retrospective study using mortality records obtained from the CDC WONDER database, specifically drawing from the Multiple Cause of Death Public Use dataset. This dataset encompasses mortality and population counts for all U.S. counties, alongside various socio-demographic parameters, including race/ethnicity and place of death [5]. Data were extracted on 2 May 2024. Our analysis centered on the trends in mortalities among older adults (>65 years old) from 1999 to 2020, where AS was implicated as the underlying cause of death using the International Statistical Classification of Diseases and Related Health Problems, 10th Revision codes (ICD-10), I06.0, I06.2, I35.0, I35.2. The same have been used in previous studies analyzing various aspects of AS [6,7,8]. As the analysis relied on anonymized data from a publicly accessible database, ethical approval was not applicable. The data were reported in compliance with the STROBE (Strengthening the Reporting of Observational Studies in Epidemiology) guidelines [9] (Supplementary Table S1).

We extracted data regarding year, population, demographics, states, urban–rural classification, and regions to analyze the temporal trends in AS mortality across various socio-demographic parameters. Demographic variables included sex, race/ethnicity, place of death, and age. Racial/ethnic groups were categorized as Hispanic or Latino, Non-Hispanic (NH) White, NH American Indian or Alaska Native, NH Black or African American, and NH Asian or Pacific Islander. These classifications rely on information recorded in death certificates, as reported by the funeral director either through information provided by the next of kin or self-reported based on observation, and have been used in the previous analyses of the WONDER dataset [10]. The location of death was categorized as home, hospice, nursing or long-term care facility, or medical facility, with the latter further classified as inpatient, outpatient, or emergency department, dead on arrival, or unknown. Urban–rural differences were assessed using the National Center for Health Statistics framework, which distinguishes metropolitan from nonmetropolitan regions. Metropolitan regions were further classified as large (populations over 1 million), medium (250,000–999,999), or small (below 250,000). Nonmetropolitan areas encompassed micropolitan regions (urban clusters with 10,000–49,999 residents) and noncore counties, which are rural areas not classified as part of a metropolitan or micropolitan area [11]. Regional designations followed the U.S. Census Bureau’s definitions of Northeast, Midwest, South, and West [11].

## 4. Statistical Analysis

Our analysis examined the trends in AS mortality from 1999 to 2020, stratified across various demographic, regional, and geographic factors. Crude mortality rates per 100,000 individuals were extracted, and age-adjusted mortality rates (AAMRs) per 100,000 individuals were calculated using the year 2000 U.S. population as the reference standard for adjustment [12]. Data points with fewer than 20 mortality events were regarded as unreliable and not included in the analysis. Annual percent changes (APCs) in AAMRs, along with their corresponding 95% confidence intervals (CIs), were calculated using the Joinpoint Regression Program version 5.4.0 to assess temporal changes in AAMRs. This method entails fitting log-linear regression models to detect significant changes in AAMR over time and has been previously used to analyze trends in mortality attributable to various diseases [13,14]. Subsequently, the weighted averages of the APCs were also calculated, the average annual percent change (AAPC) with their 95% confidence intervals, which provides a summary of the mortality trends over the complete duration of the study. All APCs/AAPCs were estimated based on AAMR (standardized to the 2000 US standard population). For subgroup analyses (sex, race/ethnicity, state, region, and urban–rural categories), AAMRs were calculated separately for each subgroup, and APCs/AAPCs were then computed independently for each stratum. Annual percent changes (APCs) were classified as rising or declining based on whether they showed a statistically significant difference from zero trend, as determined by a two-tailed *t*-test with a significance threshold of *p* < 0.05. To ensure that the COVID-19 pandemic did not distort long-term trends, a sensitivity analysis was performed by excluding data from 2020, confirming the robustness of the overall findings.

## 5. Results

**Figure 1:**
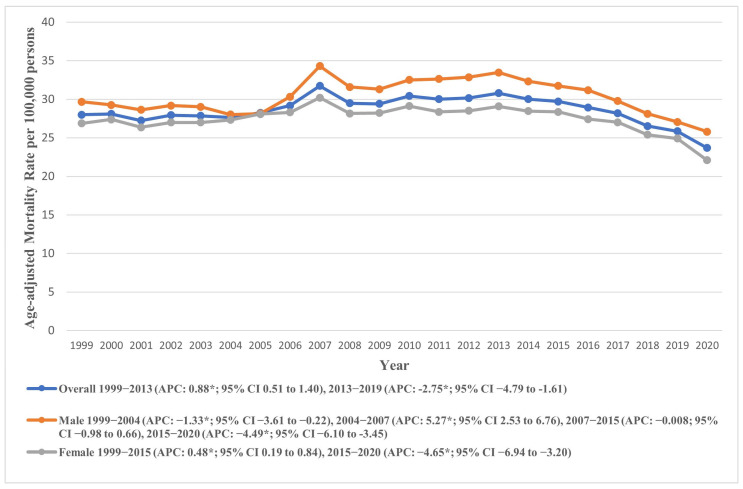
National trends in overall and sex-specific age-adjusted mortality rates due to AS in the United States from 1999 to 2020; * denotes years in which the annual percentage change (APC) differed significantly from 0 at α = 0.05.

A total of 267,515 deaths were recorded amongst older adults from 1999–2020, in which AS was implicated as the underlying cause of death. Profound sex-based and ethnic disparities were evident: 61.3% of the deaths occurred in females, and 91.7% occurred in the NH White population, followed by 3.6% amongst NH Black or African Americans (Table 1). Racial/ethnic data were available for 99.84% of the recorded deaths. The overall AAMR for the entire study duration was 28.62 (95% CI: 28.51 to 28.73). Location of death information was available for 99.8% mortalities, with the largest proportion of deaths (39.31%) occurring in medical inpatient care facilities, followed by decedents’ homes (25.78%) and nursing home/long-term care facilities (21.90%) (Figure 1) (Supplementary Table S2).

## 6. Annual Trends for AAMR

The AAMR for AS mortality registered an overall decline during the study period from 28.00 in 1999 to 23.69 in 2020 (AAPC: −0.55, 95% CI: −0.82 to −0.28). Temporal analysis revealed a continuous rise in AAMR from 1999 to 2014 (APC: 0.80, 95% CI: 0.45 to 1.25), followed by a significant decline from 2014 to 2020 (APC: −3.85, 95% CI: −5.68 to −2.65). As a sensitivity analysis, we excluded data from the year 2020 to account for potential biases related to the COVID-19 pandemic. The results remained directionally consistent, indicating that the observed mortality trends were not driven by the inclusion of pandemic-era data (Supplementary Figure S1). These trends are visually represented in Figure 1, with detailed data provided in Supplementary Tables S3 and S4.

## 7. Demographic Trends in AAMR

### 7.1. Sex-Based Trends

Males exhibited a higher AAMR (overall AAMR: 30.35, 95% CI: 30.17 to 30.54) throughout the study duration compared to females (overall AAMR: 27.42, 95% CI: 27.29 to 27.55). Despite lower AAMRs, the total number of deaths in females (164,104) were much higher in females as compared to males (103,411). In males, the AAMR recorded an initial decline from 1999 to 2004 (APC: −1.33, 95% CI: −3.61 to −0.22), followed by a sharp rise from 2004 to 2007 (APC: 5.28, 95% CI: 2.53 to 6.76). The AAMR then remained relatively stable from 2007 to 2015 (APC: −0.008, 95% CI: −0.98 to 0.66), after which it declined until 2020 (APC: −4.49, 95% CI: −6.10 to −3.45).

Conversely, the female AAMR rose from 1999 to 2015 (APC: 0.48, 95% CI: 0.19 to 0.84) and then declined markedly till 2020 (APC: −4.65, 95% CI: −6.94 to −3.20). These trends are visually represented in Figure 1, with detailed data provided in Supplementary Tables S3 and S4.

### 7.2. Racial/Ethnic Trends

We also analyzed racial/ethnic trends in AAMR across the study period, revealing a striking contrast among the different groups. NH Whites had higher AAMR as compared to other groups. The overall AAMR of NH White was 31.61 (95% CI: 31.48 to 31.73), nearly twice as high as those for other races. This was followed by NH American Indian/Alaskan Natives (overall AAMR: 16.62, 95% CI: 15.30 to 17.94), Hispanic or Latinos (overall AAMR: 14.51, 95% CI: 14.20 to 14.83), and NH Black or African American individuals (overall AAMR: 13.30, 95% CI: 13.03 to 13.57). The lowest overall AAMR was observed among NH Asian or Pacific Islanders (overall AAMR: 11.50, 95% CI: 11.12 to 11.89).

**Figure 2:**
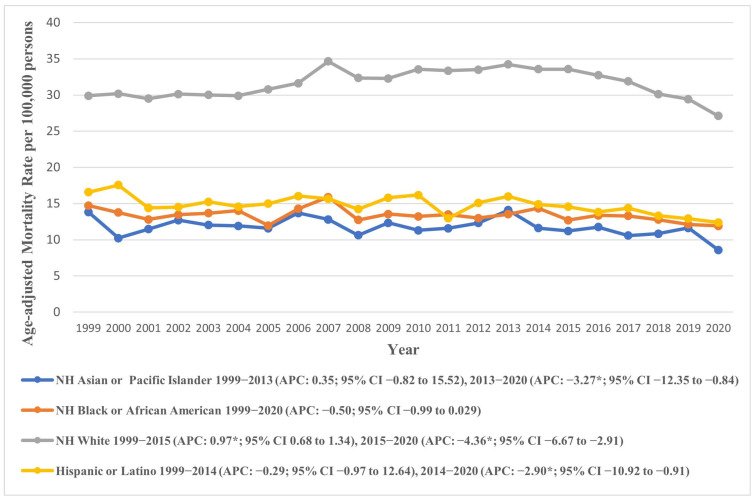
Age-adjusted mortality rates attributable to AS across racial/ethnic groups in the United States from 1999 to 2020; * denotes years in which the annual percentage change (APC) differed significantly from 0 at α = 0.05.

The AAMRs depicted an increasing trend across most groups from 1999 to the 2013–2015 period, followed by a subsequent decline until 2020. Specifically, a non-significant rise was observed in the AAMR of NH Whites from 1999 to 2015 (APC: 0.97, 95% CI: 0.68 to 1.34), followed by a steep decline till 2020 (APC: −4.36, 95% CI: −6.67 to −2.91), culminating in an overall slight decline across the study period (AAPC: −0.32, 95% CI: −0.58 to −0.05). Other groups followed similar trends except for NH Blacks, whose AAMRs varied markedly across the study group, exhibiting an overall decline (APC: −0.50, 95% CI: −0.99 to 0.03). These trends are visually represented in Figure 2, with detailed data provided in Supplementary Tables S3 and S5.

### 7.3. Geographical and Regional Trends Across the U.S.

#### 7.3.1. States

**Figure 3:**
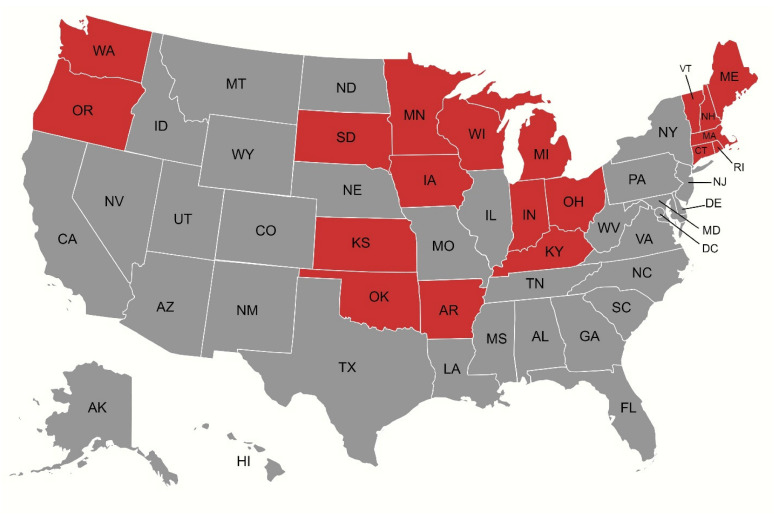
Map illustrating states (depicted in red) that experienced a net increase in age-adjusted mortality rates (AAMRs) over the study period, as reflected by their average annual percentage changes (AAPCs).

Prominent variations were observed across U.S. states, with AAMRs ranging from 60.55 (95% CI: 59.19 to 61.92) in Oregon to 17.23 (95% CI: 16.33 to 18.13) in Mississippi. Temporal trends in AAMR also depicted marked variations. While most states experienced an overall decline across the study period, several—including Arkansas, Connecticut, Indiana, Iowa, Kansas, Kentucky, Maine, Massachusetts, Michigan, Minnesota, New Hampshire, Ohio, Oklahoma, Oregon, Rhode Island, South Dakota, Vermont, Washington, and Wisconsin—showed an increase in AAMR according to their AAPCs (Figure 3). Among them, South Dakota witnessed the highest rise (AAPC: 2.74, 95% CI: 1.75 to 3.90), with the AAMR soaring from 17.53 (95% CI: 10.71 to 27.07) in 1999 to 33.17 (95% CI: 24.62 to 43.73) in 2020, followed by Maine (AAPC: 2.48, 95% CI: 1.15 to 5.16) where an AAMR of 32.93 (95% CI: 25.13 to 42.38) in 1999 rose to 51.66 (95% CI: 43.04 to 60.29) in 2020.

Other states saw a decline in their AAMRs, with Georgia recording the largest decline (AAPC: −2.84, 95% CI: −3.61 to −2.01). In Georgia, the AAMR saw an initial rise from 1999 to 2004 (APC: 0.90, 95% CI: −1.76 to 11.61), after which it declined till 2017 (APC: −2.50, 95% CI: −3.77 to −1.17). In the subsequent period between 2017 and 2020, a further profound decline was observed with an APC of −10.12 (95% CI: −18.94 to −5.05).

**Figure 4:**
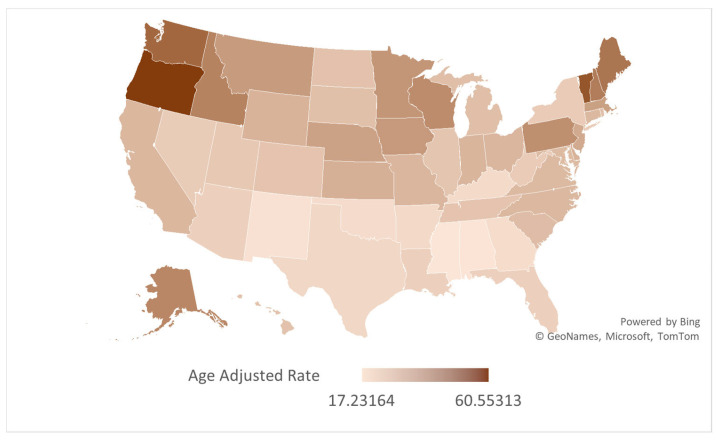
Map depicting state-wise variations in AS-associated AAMRs in older adults per 100,000 in the United States, 1999 to 2020.

Generally, an increasing trend was observed in the AAMR till the 2013–2014 period, after which a subsequent decline lasted till 2020. There were several exceptions in which the AAMR demonstrated contrasting trends; in Alabama, the AAMR decreased throughout the study with an initially gentle slope till 2014 (APC: −0.62, 95% CI: −1.86 to 11.12), followed by a marked reduction till 2020 (APC: −7.07, 95% CI: −22.93 to −2.46). The temporal variations in AAMR across states are visually represented in Figure 4, with detailed data provided in Supplementary Tables S3 and S6.

#### 7.3.2. Regions

Our analysis across the four U.S. Census regions according to the U.S. Census Bureau’s Classification revealed the Northeast (overall AAMR: 32.087, 95% CI: 31.83 to 32.34) and West (overall AAMR: 32.092, 95% CI: 31.84 to 32.34) as regions with the highest AAMRs. In contrast, the Southern region recorded the lowest AAMR (23.06, 95% CI: 22.90 to 23.23).

**Figure 5:**
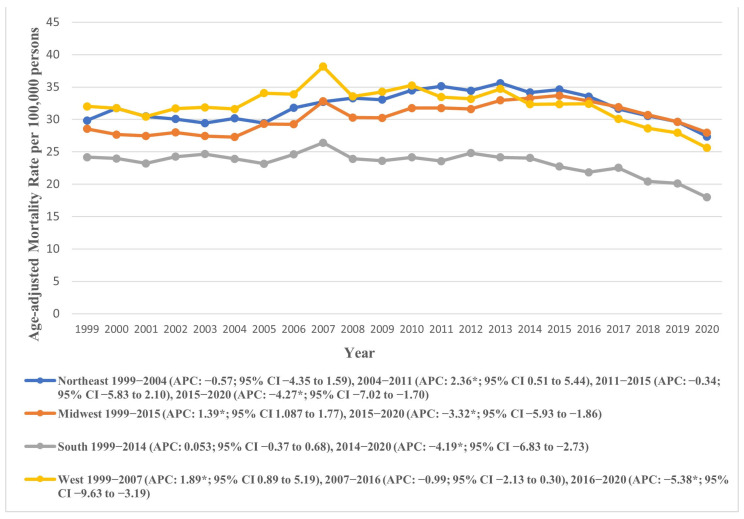
Age-adjusted mortality rates attributable to AS across regions in the United States from 1999 to 2020; * denotes years in which the annual percentage change (APC) differed significantly from 0 at α = 0.05.

Across the study period, the AAPC revealed a decline in AAMRs across all regions except for the Midwest, which saw a rise (AAPC: 0.25, 95% CI: −0.002 to 0.53). The temporal variations in AAMR across census regions are visually represented in Figure 5, with detailed data provided in Supplementary Tables S3 and S7.

#### 7.3.3. Urban–Rural Status

**Figure 6:**
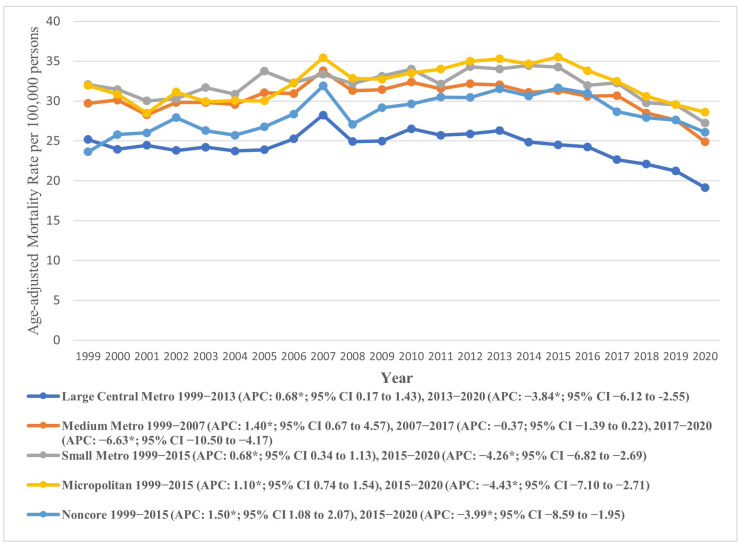
Age-adjusted mortality rates attributable to AS across urban and rural populations in the United States from 1999 to 2020; * denotes years in which the annual percentage change (APC) differed significantly from 0 at α = 0.05.

Marked variations in AAMR were also observed across urban–rural status, with micropolitan areas exhibiting profoundly higher AAMRs (overall AAMR: 32.28, 95% CI: 31.92 to 32.65), compared to large central metropolitan areas (overall AAMR: 24.32, 95% CI: 24.13 to 24.51). The AAMRs for both micropolitan (APC: 1.10, 95% CI: 0.74 to 1.54) and small metropolitan areas (APC: 0.68, 95% CI: 0.34 to 1.13) increased till 2015, with a subsequent decline till 2020 (micropolitan APC: −4.43, 95% CI: −7.10 to −2.71), (small metropolitan APC: −4.26, 95% CI: −6.82 to −2.69). Similar trends were observed in other areas. Notably, the AAMRs of all areas recorded an overall decline at the end of the study period, apart from Noncore (non-metropolitan) areas, with a rise from 23.63 (95% CI: 21.91 to 25.35) in 1999 to 26.08 (95% CI: 24.46 to 27.70) in 2020 (AAPC: 0.16, 95% CI: −0.20 to 0.56). These trends are depicted in Figure 6 and detailed in Supplementary Tables S3 and S8.

## 8. Discussion

This analysis spanning two decades of mortality data revealed several compelling insights. Overall, mortality rates from AS remained relatively stable with a slight increase from 1999 to 2014, after which they declined. The initial rise in mortality may be a result of improved disease detection, potentially driven by increased echocardiography utilization and evolving diagnostic coding practices [15]. Our findings are in line with data from 2008 to 2017, looking at patients above the age of 45 who had AS listed as an underlying cause of death [8].

TAVR was first approved in 2011 for patients with severe AS with prohibitive operative risk. In 2012, its use was expanded to high-surgical-risk patients and, in 2015, to “valve-in-valve” procedures [16]. In 2016, it was approved for use in intermediate-risk patients, and after the PARTNER-3 trial in 2019, the indication was expanded to include low-risk patients [17]. The expanding use of TAVR, with its demonstrated improvements in mortality, stroke, duration of hospitalization, and 30-day readmission compared to surgical AVR, correlates with the decreasing mortality from AS in 2015–2020 [18].

The link between TAVR practice developments and AS mortality trends cannot be assumed to be causal. TAVR uptake may indicate other aspects of high-value care that could improve mortality. Additionally, TAVR and surgical AVR rates are inconsistently collected from various sources, and other factors influence AS mortality, such as surgical AVR experience, AS diagnosis frequency, and the prevalence and management of risk factors (e.g., chronic renal disease, hypercholesterolemia, diabetes, smoking). These risk factors significantly overlap with those for atherosclerotic cardiovascular disease, and care has continued to improve for these conditions in the past few years. Moreover, the management of comorbidities among patients undergoing TAVR has been continuously improving, with broader adoption of contemporary preventive therapies such as SGLT2 inhibitors, which may have contributed to the observed mortality decline [19,20].

Despite the overall improvement in AAMR, the data does show some concerning trends. While males had a higher AAMR compared to females, the total deaths in females were more than in males. The literature shows that women are diagnosed with AS in later stages, are more symptomatic on diagnosis, have fewer comorbidities, and are managed conservatively for longer compared to men, despite having higher left atrial volumes and pulmonary pressures [21]. Though women have a longer average life expectancy compared to men, women with severe AS have a higher mortality rate than men of the same age [21]. Data show female sex is associated with improved survival at 1 year after TAVR [22]. Addressing sex disparities in the treatment and outcomes of severe AS is crucial to ensuring equitable care.

Mortality due to AS remained largely unchanged among NH American Indian/Alaskan, Hispanic/Latino, NH Black/African American, and NH Pacific islanders, in contrast to the decline observed in NH white patients. This is evidenced by the steep decline in AAMR noted in NH Whites (APC: −4.36, 95% CI: −6.67 to −2.91) from 2015–2020. This is in comparison to the APC for other racial and ethnic groups.

It is likely that the incidence of AS is underreported in the non-white cohort. Racial disparities in this cohort have been extensively reported in the literature. A racial and ethnic gap between TAVR utilization rates has already been documented [23]. The underlying causes of racial and ethnic disparities in TAVR utilization remain incompletely understood and are likely multifactorial. Potential contributors include physician decision-making and implicit bias, variations in patient preferences and health literacy, differences in access to subspecialty care, and the broader influence of structural inequities within the healthcare system [24]. Moreover, disparities have been observed in diagnostic coding practices for AS, with Non-Hispanic Black and Non-Hispanic Asian patients being less likely than their Non-Hispanic White counterparts to receive an ICD code confirming AS after initial transthoracic echocardiography. This distinction is critical, as the presence of a diagnostic code often determines eligibility for further evaluation and intervention, including TAVR [25]. Additional research is warranted to better delineate the factors underlying these racial differences in diagnosis, treatment, and outcomes.

Our analysis uncovered profound regional and geographic variations in AS mortality trends. Most states had an initial rise in AAMRs until 2013–2015, followed by a decline until 2020, culminating in an overall decline in AAMRs across the study period. However, 19 states deviated from this trend, recording increases in AAMR over the course of the study. These states were mostly located in the Midwest and Northeast regions, with South Dakota and Maine experiencing the highest increases in AAMRs.

Although the precise reasons behind these divergent trends remain unclear, they are likely influenced by a multitude of factors. Certain states, including Maine, Vermont, Iowa, New Hampshire, and Oregon, have comparatively older populations and higher proportions of White populations, which may partly contribute to increased incidence and mortality in these regions. Additionally, the harsh winter climates in some northern and midwestern states may exacerbate cardiovascular conditions, potentially contributing to increased mortality rates [26]. Furthermore, states with higher percentages of rural populations often lack robust healthcare infrastructures and have higher uninsured rates, creating barriers to timely and specialized care. Lifestyle factors such as the high prevalence of obesity, hypertension, and smoking—particularly pervasive in some Midwestern and Southern states—also elevate the risk of developing AS, contributing to higher incidence in some states [27,28].

Among the four U.S. Census regions, the Northeast and West exhibited the highest overall AAMRs, both around 32.09, while the South had the lowest at 23.06. The AAMR generally declined across all regions during the study period, except for the Midwest, which saw a slight increase. Urban–rural trends showed that micropolitan areas had notably higher AAMRs (32.28) compared to large central metropolitan areas (24.32). Both micropolitan and small metropolitan areas experienced an initial increase in AAMR until 2015, followed by a significant decline. In contrast, noncore (non-metropolitan) areas showed a continuous rise in AAMR from 1999 to 2020. Socioeconomic factors, including higher income, better education, and broader insurance coverage, are more prevalent in regions like the Northeast, contributing to lower AS mortality rates. Lifestyle and health behaviors, such as higher prevalence of chronic diseases and smoking, further impact AS outcomes, especially in rural and certain Midwest and Southern states. Variations in healthcare policies, with some regions adopting advanced treatments like TAVR more rapidly, also influence mortality rates.

## 9. Limitations

Our study utilized data from the CDC WONDER database and identified deaths that listed AS as the primary or underlying cause of death using ICD-10 codes. However, reliance on administrative coding introduces the potential for misclassification bias, as inaccuracies in death certification or coding practices may lead to underreporting or overestimation of AS-related mortality. Additionally, the database lacks detailed clinical information, including laboratory parameters, imaging findings, treatment modalities such as TAVR or surgical valve replacement, and medication use. It also does not include socioeconomic variables, comorbidity burden, or access-to-care indicators, all of which may substantially affect mortality outcomes and contribute to observed demographic or regional disparities. These limitations may, therefore, influence the precision of our findings and should be considered when interpreting the results.

## 10. Conclusions

Our study highlights persistent disparities in AS-related mortality across demographic and geographic groups. Mortality rates have declined in recent years, potentially reflecting advances in diagnostic practices and broader adoption of TAVR. Notably, however, sex-, race-, and region-based differences remain evident and may be widening over time. Further research is needed to elucidate the drivers of these disparities and to develop targeted interventions that ensure more equitable access to diagnosis and treatment.

## Figures and Tables

**Table 1:** AS deaths, stratified by sex and race, in the United States, 1999 to 2020.

Year	Overall	Male	Female	NH White	NH Black or African American	NH American Indian or Alaskan Native	NH Asian or Pacific Islander	Hispanic or Latino	Population
1999	9572	3462	6110	8846	385	18	83	219	34,797,841
2000	9743	3439	6304	9038	364	18	67	239	34,991,753
2001	9615	3461	6154	8952	339	15	80	214	35,290,291
2002	9961	3575	6386	9252	356	20	95	223	35,522,207
2003	10,105	3647	6458	9364	365	19	96	250	35,863,529
2004	10,175	3574	6601	9419	378	18	102	251	36,203,319
2005	10,635	3702	6933	9901	330	21	106	267	36,649,798
2006	11,272	4127	7145	10,402	403	21	133	300	37,164,107
2007	12,566	4803	7763	11,627	461	21	130	319	37,825,711
2008	11,939	4555	7384	11,092	376	29	118	309	38,777,621
2009	12,179	4653	7526	11,223	414	27	145	359	39,623,175
2010	12,865	4940	7925	11,884	415	25	140	383	40,267,984
2011	13,113	5183	7930	12,129	436	35	159	334	41,394,141
2012	13,509	5373	8136	12,397	438	36	180	420	43,145,356
2013	14,134	5681	8453	12,911	472	37	226	470	44,704,074
2014	14,066	5664	8402	12,804	522	39	203	468	46,243,211
2015	14,297	5731	8566	13,024	480	39	211	494	47,760,852
2016	14,202	5783	8419	12,871	526	35	236	497	49,244,195
2017	14,070	5682	8388	12,693	541	38	228	546	50,858,679
2018	13,556	5523	8033	12,170	543	41	247	533	52,431,193
2019	13,431	5485	7946	12,032	526	38	282	538	54,058,263
2020	12,510	5368	7142	11,167	536	38	220	538	55,659,365
Total	267,515	103,411	164,104	245,198	9606	628	3487	8171	928,476,665

## Data Availability

All data supporting the findings of this study are included within the manuscript and its accompanying Supplementary File. The dataset is publicly accessible through the CDC WONDER Multiple Cause of Death database at: https://wonder.cdc.gov/mcd.html (accessed on 10 November 2025).

## Supplementary Materials

The following supporting information can be downloaded at: https://www.mdpi.com/article/10.3390/jcm14238276/s1, Figure S1: Trends in age-adjusted mortality rates (AAMR) due to aortic stenosis in the United States from 1999 to 2020, with Joinpoint regression identifying significant shifts in temporal trends. * indicates a statistically significant annual percent change (APC) at α = 0.05; Figure S2: Sensitivity analysis of age-adjusted mortality rates (AAMR) due to aortic stenosis from 1999 to 2019, excluding the year 2020 to minimize potential confounding effects of the COVID-19 pandemic. Joinpoint regression was used to detect significant changes in trend; * indicates a statistically significant APC at α = 0.05; Table S1: STROBE Statement; Table S2: AS related mortality stratified by place of death in the United States from 1999 to 2020; Table S3: Annual percent change (APC) of AS-associated age-adjusted mortality rates per 100,000 in the United States, 1999 to 2020; Table S4: Overall and sex-stratified AS-associated age-adjusted mortality rates per 100,000 in the United States, 1999 to 2020; Table S5: Racial trends in AS-associated age-adjusted mortality rates per 100,000, in the United States, 1999 to 2020; Table S6: AS-associated age-adjusted mortality rates per 100,000, stratified by State in the United States, 1999 to 2020; Table S7: AS-associated age-adjusted mortality rates per 100,000, stratified by Census Region in the United States, 1999 to 2020; Table S8: AS-associated age-adjusted mortality rates per 100,000, stratified by urban–rural status in the United States, 1999 to 2020.
