# Supplementary material for: Trends in Aortic Stenosis Mortality Among Older Adults in the United States from 1999 to 2020
Source: J Clin Med. 2025 Nov 21;14(23):8276. doi: 10.3390/jcm14238276 (PMC12693473; doi:10.3390/jcm14238276)
Supplement: Supplementary file 1 [file jcm-14-08276-s001.zip › jcm-3930645-supplementary.pdf]

Supplementary File

Trends in Aortic Stenosis mortality among older adults in the United States from 1999 to 2020

Authors:

Muhammad Ahmad [a]*, Salman Zahid [b]*, Mustafa Shehzad [c], Dawood Shehzad [d], Evan Shalen [b], Hind Rahmouni [b], Muhammad Raza [e], Craig Basman [f], Marian Vandyck-Acquah [f], Ryan Kaple [f]

Dr. Ahmad and Dr. Zahid contributed equally and are primary co-authors.

[a] Department of Medicine, Khyber Medical College, Peshawar, Pakistan muhammadahmad.mail44@gmail.com

[b] Department of Cardiovascular Medicine, Knight Cardiovascular Institute, Oregon Health and Science University, Portland, OR, USA zahidsa@ohsu.edu, shalen@ohsu.edu, rahmouni@ohsu.edu

[c] Department of Internal Medicine, Hackensack University Medical Center, Hackensack, NJ, USA mustafa.shehzad@hmhn.org

[d] Department of Internal Medicine, University of South Dakota, Sanford School of Medicine, Sioux Falls, SD, USA Daud.shehzad@usd.edu

[e] Department of Cardiology, Deborah Heart and Lung Center, Brown Mills, NJ, USA razam@deborah.org

[f] Department of Cardiology, Hackensack University Medical Center, Hackensack Meridian School of Medicine, Hackensack, NJ, USA craig.basman@hmhn.org, marian.vandyck-acquah@hmhn.org, ryan.kaple@hmhn.org

Corresponding author:

Ryan Kaple, MD

Director Structural & Congenital Heart Program

Hackensack University Medical Center, Hackensack Meridian School of Medicine

30 Prospect Ave, Hackensack, NJ 07601

ryan.kaple@hmhn.org

Content

Figures: 2

Tables: 8

Abbreviations:

AAMR: Age Adjusted Mortality Rate

NH: Non-Hispanic

APC: Average Percentage Change

AAPC: Average Annual Percentage Change

Figures:

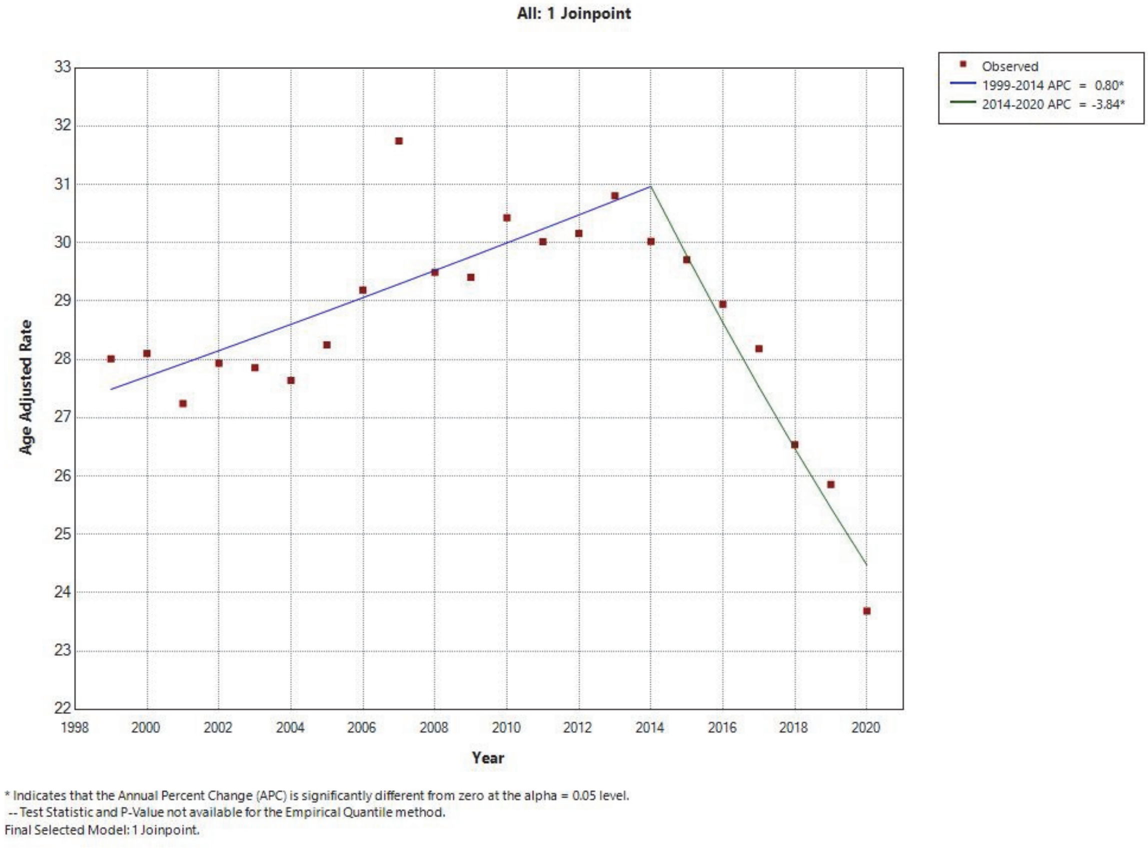

Figure S1: Trends in age-adjusted mortality rates (AAMR) due to aortic stenosis in the United States from 1999 to 2020, with Joinpoint regression identifying significant shifts in temporal trends. * indicates a statistically significant annual percent change (APC) at $\alpha = 0.05$.

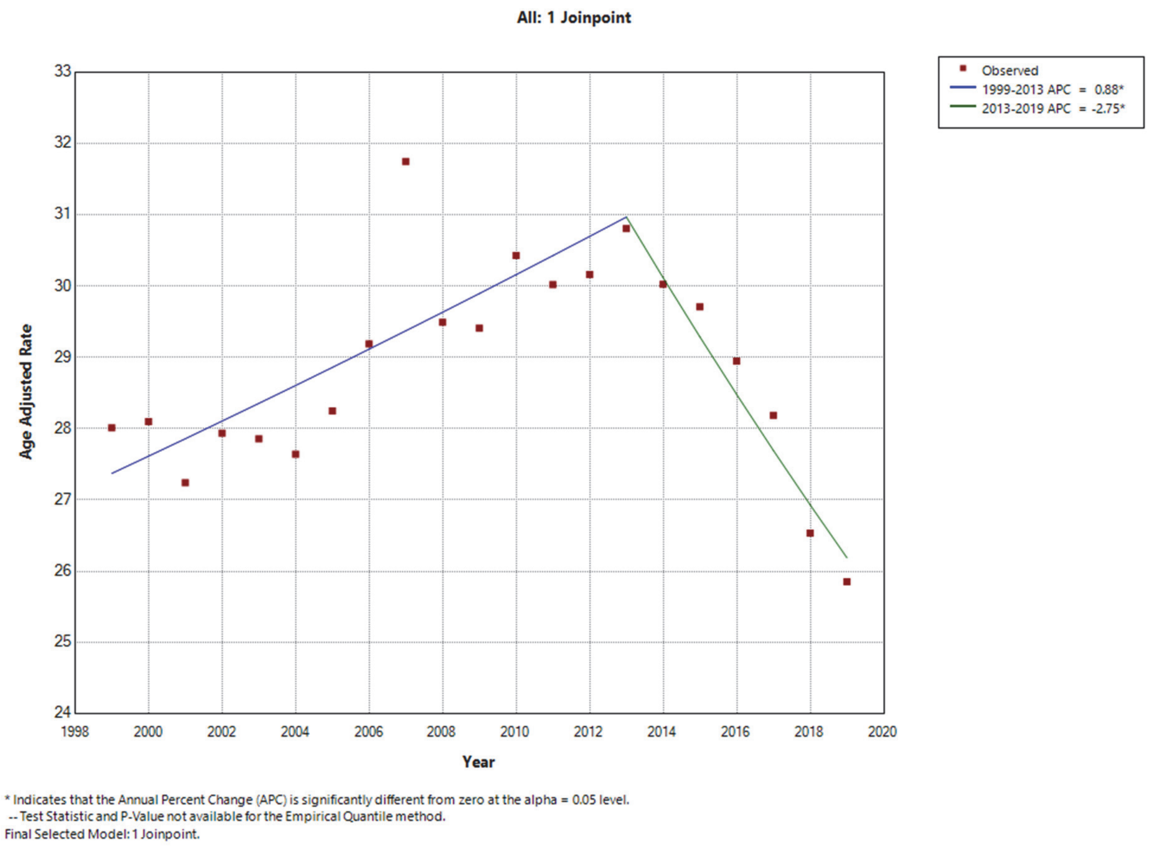

Figure S2: Sensitivity analysis of age-adjusted mortality rates (AAMR) due to aortic stenosis from 1999 to 2019, excluding the year 2020 to minimize potential confounding effects of the COVID-19 pandemic. Joinpoint regression was used to detect significant changes in trend; * indicates a statistically significant APC at α = 0.05.

Tables

	Item No	Recommendation	Page No
**Title and abstract**	1	(*a*) Indicate the study's design with a commonly used term in the title or the abstract	1-2
		(*b*) Provide in the abstract an informative and balanced summary of what was done and what was found	2
**Introduction**			
Background/rationale	2	Explain the scientific background and rationale for the investigation being reported	3
Objectives	3	State specific objectives, including any prespecified hypotheses	3
**Methods**			
Study design	4	Present key elements of study design early in the paper	3-4
Setting	5	Describe the setting, locations, and relevant dates, including periods of recruitment, exposure, follow-up, and data collection	4
Participants	6	(*a*) Give the eligibility criteria, and the sources and methods of selection of participants. Describe methods of follow-up	4
		(*b*) For matched studies, give matching criteria and number of exposed and unexposed	
Variables	7	Clearly define all outcomes, exposures, predictors, potential confounders, and effect modifiers. Give diagnostic criteria, if applicable	4-5
Data sources/ measurement	8*	For each variable of interest, give sources of data and details of methods of assessment (measurement). Describe comparability of assessment methods if there is more than one group	4-5
Bias	9	Describe any efforts to address potential sources of bias	NA
Study size	10	Explain how the study size was arrived at	NA
Quantitative variables	11	Explain how quantitative variables were handled in the analyses. If applicable, describe which groupings were chosen and why	5-6
Statistical methods	12	(*a*) Describe all statistical methods, including those used to control for confounding	5-6

		(*b*) Describe any methods used to examine subgroups and interactions	NA
		(*c*) Explain how missing data were addressed	5
		(*d*) If applicable, explain how loss to follow-up was addressed	NA
		(*e*) Describe any sensitivity analyses	
**Results**			
Participants	13*	(a) Report numbers of individuals at each stage of study—eg numbers potentially eligible, examined for eligibility, confirmed eligible, included in the study, completing follow-up, and analysed	6
		(b) Give reasons for non-participation at each stage	NA
		(c) Consider use of a flow diagram	NA
Descriptive data	14*	(a) Give characteristics of study participants (eg demographic, clinical, social) and information on exposures and potential confounders	6
		(b) Indicate number of participants with missing data for each variable of interest	NA
		(c) Summarise follow-up time (eg, average and total amount)	NA
Outcome data	15*	Report numbers of outcome events or summary measures over time	6-10
Main results	16	(*a*) Give unadjusted estimates and, if applicable, confounder-adjusted estimates and their precision (eg, 95% confidence interval). Make clear which confounders were adjusted for and why they were included	6-10
		(*b*) Report category boundaries when continuous variables were categorized	NA
		(*c*) If relevant, consider translating estimates of relative risk into absolute risk for a meaningful time period	NA
Other analyses	17	Report other analyses done—eg analyses of subgroups and interactions, and sensitivity analyses	6-10
**Discussion**			
Key results	18	Summarise key results with reference to study objectives	10

Limitations	19	Discuss limitations of the study, taking into account sources of potential bias or imprecision. Discuss both direction and magnitude of any potential bias	14
Interpretation	20	Give a cautious overall interpretation of results considering objectives, limitations, multiplicity of analyses, results from similar studies, and other relevant evidence	10-14
Generalisability	21	Discuss the generalisability (external validity) of the study results	10-14
**Other information**			
Funding	22	Give the source of funding and the role of the funders for the present study and, if applicable, for the original study on which the present article is based	

Supplementary Table S1. STROBE Statement- This study adhered to the STROBE (Strengthening the Reporting of Observational Studies in Epidemiology) guidelines for rigorous reporting standards.

Year	Inpatient	Outpatient	Dead on Arrival	Decedent's home	Nursing home/Long term care
**1999**	4585	510	81	1914	2147
**2000**	4605	458	74	2042	2203
**2001**	4481	439	48	2026	2187
**2002**	4430	488	58	2145	2380
**2003**	4395	410	52	2339	2450
**2004**	4428	421	55	2362	2325
**2005**	4350	443	39	2611	2566
**2006**	4648	458	40	2815	2632
**2007**	5217	575	51	3003	2864
**2008**	4834	478	36	2888	2731
**2009**	4696	497	30	3038	2759
**2010**	4925	522	40	3405	2861
**2011**	5020	528	31	3354	2998
**2012**	5021	530	31	3706	2880
**2013**	5254	522	36	3782	3017
**2014**	5089	518	21	3841	2978
**2015**	5285	471	21	3851	3082
**2016**	5013	463	22	4016	2946
**2017**	5071	455	22	3899	2916
**2018**	4856	439	21	3873	2745
**2019**	4767	429	20	3794	2766
**2020**	4187	410	12	4261	2145
**Total**	105157	10464	841	68965	58578

Supplementary Table S2. AS related mortality stratified by place of death in the United States from 1999 to 2020.

Year Interval	APC (95% CI)
**Overall**	
1999-2014	0.80 (0.45 to 1.25)
2014-2020	-3.84 (-5.68 to -2.65)
**Male**	
1999-2004	-1.33 (-3.61 to -0.22)
2004-2007	5.28 (2.53 to 6.76)
2007-2015	-0.01 (-0.98 to 0.66)
2015-2020	-4.49 (-6.10 to -3.45)
**Female**	
1999-2015	0.48 (0.19 to 0.84)
2015-2020	-4.65 (-6.94 to -3.20)
**Race/Ethnicity**	
**NH Asian or Pacific Islander**	
1999-2013	0.35 (-0.82 to 15.52)
2013-2020	-3.27 (-12.35 to -0.84)
**NH Black**	
1999-2020	-0.50 (-0.99 to 0.03)
**NH White**	
1999-2015	0.97 (0.68 to 1.34)
2015-2020	-4.36 (-6.67 to -2.91)
**Hispanic or Latino**	
1999-2014	-0.29 (-0.97 to 12.64)
2014-2020	-2.90 (-10.92 to -0.91)
**Urbanization**	
**Large Central Metro**	
1999-2013	0.68 (0.17 to 1.43)
2013-2020	-3.84 (-6.12 to -2.55)
**Small metropolitan**	
1999-2015	0.68 (0.34 to 1.13)
2015-2020	-4.26 (-6.82 to -2.69)
**Micropolitan (Nonmetro)**	
1999-2015	1.10 (0.74 to 1.54)
2015-2020	-4.43 (-7.10 to -2.71)
**Noncore (Nonmetro)**	
1999-2015	1.50 (1.08 to 2.07)
2015-2020	-3.99 (-8.59 to -1.95)
**Census Region**	
**Northeast**	

1999-2004	-0.57 (-4.35 to 1.59)
2004-2011	2.36 (0.51 to 5.44)
2011-2015	-0.34 (-5.83 to 2.10)
2015-2020	-4.27 (-7.02 to -1.70)
**Midwest**	
1999-2015	1.39 (1.09 to 1.77)
2015-2020	-3.32 (-5.93 to -1.86)
**South**	
1999-2014	0.05 (-0.37 to 0.68)
2014-2020	-4.19 (-6.83 to -2.73)
**West**	
1999-2007	1.89 (0.89 to 5.19)
2007-2016	-0.99 (-2.13 to 0.30)
2016-2020	-5.38 (-9.63 to -3.19)

Supplementary Table S3. Annual percent change (APC) of AS associated Age-Adjusted Mortality Rates per 100,000 in the United States, 1999 to 2020

	Age Adjusted Rate (95% CI)		
**Year**	**Male**	**Female**	**Overall**
**1999**	29.66 (28.66 to 30.67)	26.87 (26.20 to 27.55)	28.01 (27.45 to 28.57)
**2000**	29.27 (28.28 to 30.26)	27.38 (26.70 to 28.06)	28.10 (27.54 to 28.66)
**2001**	28.63 (27.66 to 29.60)	26.36 (25.70 to 27.03)	27.24 (26.70 to 27.79)
**2002**	29.18 (28.21 to 30.15)	26.99 (26.32 to 27.65)	27.93 (27.39 to 28.48)
**2003**	29.02 (28.06 to 29.97)	26.99 (26.33 to 27.65)	27.86 (27.31 to 28.40)
**2004**	28.02 (27.09 to 28.95)	27.33 (26.66 to 27.99)	27.64 (27.10 to 28.18)
**2005**	28.14 (27.23 to 29.06)	28.09 (27.42 to 28.75)	28.25 (27.71 to 28.78)
**2006**	30.34 (29.40 to 31.27)	28.31 (27.65 to 28.97)	29.18 (28.65 to 29.72)
**2007**	34.30 (33.33 to 35.28)	30.20 (29.53 to 30.88)	31.74 (31.19 to 32.30)
**2008**	31.60 (30.68 to 32.52)	28.16 (27.51 to 28.81)	29.49 (28.96 to 30.02)
**2009**	31.30 (30.40 to 32.20)	28.23 (27.58 to 28.87)	29.41 (28.88 to 29.93)
**2010**	32.52 (31.61 to 33.43)	29.12 (28.47 to 29.77)	30.43 (29.90 to 30.95)
**2011**	32.63 (31.74 to 33.52)	28.37 (27.74 to 29.00)	30.02 (29.50 to 30.53)
**2012**	32.86 (31.98 to 33.75)	28.52 (27.89 to 29.15)	30.16 (29.65 to 30.67)
**2013**	33.47 (32.60 to 34.34)	29.09 (28.46 to 29.72)	30.81 (30.29 to 31.32)
**2014**	32.33 (31.48 to 33.17)	28.47 (27.85 to 29.09)	30.02 (29.52 to 30.52)
**2015**	31.73 (30.90 to 32.55)	28.36 (27.75 to 28.97)	29.71 (29.22 to 30.20)
**2016**	31.19 (30.38 to 32.00)	27.43 (26.83 to 28.02)	28.94 (28.47 to 29.42)
**2017**	29.78 (29.00 to 30.56)	27.01 (26.42 to 27.60)	28.18 (27.71 to 28.65)
**2018**	28.13 (27.39 to 28.88)	25.40 (24.84 to 25.97)	26.54 (26.09 to 26.98)
**2019**	27.06 (26.34 to 27.78)	24.92 (24.36 to 25.47)	25.85 (25.41 to 26.29)

**2020**	25.79 (25.10 to 26.49)	22.11 (21.59 to 22.62)	23.69 (23.27 to 24.10)
**Total**	30.35 (30.17 to 30.54)	27.42 (27.29 to 27.55)	28.62 (28.51 to 28.73)

Supplementary Table S4. Overall and Sex-Stratified AS associated Age-Adjusted Mortality Rates per 100,000 in the United States, 1999 to 2020

Year	NH Asian or Pacific Islander	NH Black or African American	NH White	Hispanic or Latino
**1999**	13.84 (10.95 to 17.24)	14.72 (13.24 to 16.19)	29.89 (29.27 to 30.51)	16.57 (14.34 to 18.79)
**2000**	10.22 (7.87 to 13.06)	13.76 (12.35 to 15.18)	30.18 (29.56 to 30.80)	17.57 (15.32 to 19.82)
**2001**	11.48 (9.06 to 14.35)	12.82 (11.45 to 14.19)	29.49 (28.87 to 30.10)	14.40 (12.44 to 16.36)
**2002**	12.72 (10.25 to 15.60)	13.45 (12.05 to 14.85)	30.12 (29.51 to 30.74)	14.51 (12.58 to 16.45)
**2003**	12.03 (9.72 to 14.72)	13.67 (12.26 to 15.07)	30.02 (29.41 to 30.63)	15.24 (13.32 to 17.15)
**2004**	11.92 (9.58 to 14.26)	14.03 (12.61 to 15.45)	29.89 (29.29 to 30.50)	14.60 (12.76 to 16.43)
**2005**	11.60 (9.37 to 13.83)	11.95 (10.66 to 13.25)	30.79 (30.18 to 31.40)	14.98 (13.16 to 16.79)
**2006**	13.70 (11.35 to 16.05)	14.28 (12.89 to 15.68)	31.64 (31.03 to 32.25)	16.03 (14.20 to 17.87)
**2007**	12.82 (10.60 to 15.03)	15.89 (14.44 to 17.35)	34.66 (34.02 to 35.29)	15.66 (13.93 to 17.40)
**2008**	10.63 (8.70 to 12.56)	12.73 (11.44 to 14.02)	32.36 (31.75 to 32.96)	14.23 (12.63 to 15.83)
**2009**	12.35 (10.33 to 14.37)	13.55 (12.24 to 14.86)	32.28 (31.68 to 32.88)	15.80 (14.15 to 17.44)
**2010**	11.31 (9.43 to 13.19)	13.22 (11.95 to 14.50)	33.56 (32.95 to 34.16)	16.17 (14.54 to 17.80)
**2011**	11.60 (9.79 to 13.41)	13.48 (12.21 to 14.75)	33.36 (32.76 to 33.96)	12.96 (11.57 to 14.36)
**2012**	12.32 (10.51 to 14.12)	12.98 (11.76 to 14.20)	33.51 (32.91 to 34.10)	15.11 (13.66 to 16.56)
**2013**	14.09 (12.25 to 15.93)	13.54 (12.31 to 14.76)	34.22 (33.62 to 34.82)	15.98 (14.53 to 17.43)
**2014**	11.61 (10.01 to 13.22)	14.35 (13.11 to 15.59)	33.57 (32.98 to 34.16)	14.89 (13.54 to 16.25)
**2015**	11.22 (9.70 to 12.74)	12.72 (11.57 to 13.86)	33.57 (32.98 to 34.15)	14.57 (13.28 to 15.86)
**2016**	11.75 (10.25 to 13.26)	13.37 (12.22 to 14.52)	32.73 (32.16 to 33.31)	13.83 (12.61 to 15.06)
**2017**	10.57 (9.20 to 11.95)	13.31 (12.18 to 14.44)	31.89 (31.33 to 32.45)	14.36 (13.15 to 15.58)
**2018**	10.85 (9.49 to 12.20)	12.78 (11.70 to 13.87)	30.12 (29.59 to 30.66)	13.32 (12.18 to 14.46)
**2019**	11.64 (10.27 to 13.00)	12.12 (11.08 to 13.17)	29.43 (28.90 to 29.96)	12.92 (11.82 to 14.01)
**2020**	8.59 (7.45 to 9.73)	11.93 (10.91 to 12.94)	27.10 (26.59 to 27.60)	12.39 (11.34 to 13.45)
**Total**	11.50 (11.12 to 11.89)	13.30 (13.03 to 13.57)	31.61 (31.48 to 31.73)	14.51 (14.20 to 14.83)

Supplementary Table S5. Racial trends in AS associated Age-Adjusted Mortality Rates per 100,000, in the United States, 1999 to 2020.

State	Age Adjusted Rate (95% CI)	Average Annual Percentage Change from 1999-2020

		(95% CI)
**Alabama**	17.39 (16.69 to 18.08)	-2.51 (-4.30 to -0.59)
**Alaska**	41.26 (37.11 to 45.41)	----
**Arizona**	22.52 (21.84 to 23.19)	-0.55 (-1.20 to 0.18)
**Arkansas**	20.20 (19.28 to 21.12)	0.95 (-0.15 to 2.23)
**California**	29.15 (28.82 to 29.48)	-1.73 (-2.13 to -1.32)
**Colorado**	25.59 (24.69 to 26.49)	-1.06 (-1.93 to -0.05)
**Connecticut**	28.73 (27.81 to 29.65)	0.64 (-0.20 to 1.58)
**Delaware**	30.03 (27.99 to 32.07)	-1.72 (-2.97 to -0.33)
**District of Columbia**	18.03 (16.02 to 20.04)	----
**Florida**	22.56 (22.22 to 22.89)	-1.59 (-2.38 to -0.85)
**Georgia**	19.56 (18.96 to 20.16)	-2.84 (-3.61 to -2.01)
**Hawaii**	26.20 (24.76 to 27.63)	-0.05 (-1.15 to 1.24)
**Idaho**	42.29 (40.34 to 44.25)	-2.13 (-3.43 to -0.77)
**Illinois**	25.43 (24.92 to 25.93)	-0.62 (-1.23 to -0.14)
**Indiana**	29.53 (28.76 to 30.30)	0.28 (-0.31 to 0.94)
**Iowa**	36.62 (35.52 to 37.71)	0.32 (-0.96 to 2.69)
**Kansas**	30.87 (29.75 to 31.98)	0.56 (-0.33 to 1.57)
**Kentucky**	20.07 (19.28 to 20.86)	0.07 (-1.35 to 1.99)
**Louisiana**	22.69 (21.84 to 23.54)	-1.58 (-2.41 to -0.75)
**Maine**	45.85 (43.95 to 47.75)	2.48 (1.15 to 5.16)
**Maryland**	28.07 (27.26 to 28.89)	-0.08 (-0.63 to 0.54)
**Massachusetts**	34.98 (34.22 to 35.74)	0.28 (-0.43 to 0.97)
**Michigan**	27.09 (26.51 to 27.66)	0.25 (-0.16 to 0.63)
**Minnesota**	37.46 (36.54 to 38.37)	0.17 (-0.40 to 0.77)
**Mississippi**	17.23 (16.33 to 18.13)	-0.37 (-1.52 to 0.87)
**Missouri**	29.26 (28.50 to 30.02)	-0.26 (-1.54 to 0.99)
**Montana**	36.11 (34.07 to 38.14)	-1.06 (-2.93 to 1.09)
**Nebraska**	34.37 (32.92 to 35.81)	-0.80 (-1.96 to 0.48)
**Nevada**	23.64 (22.40 to 24.88)	-0.54 (-1.94 to 1.22)
**New Hampshire**	44.03 (42.01 to 46.05)	0.07 (-0.83 to 1.14)
**New Jersey**	32.62 (31.96 to 33.27)	-1.41 (-2.13 to -0.84)
**New Mexico**	18.18 (17.08 to 19.28)	-1.07 (-3.84 to 2.69)
**New York**	23.89 (23.51 to 24.27)	-0.65 (-0.95 to -0.35)
**North Carolina**	28.84 (28.19 to 29.49)	-0.92 (-1.26 to -0.55)
**North Dakota**	26.21 (24.24 to 28.19)	----
**Ohio**	29.20 (28.66 to 29.75)	0.03 (-0.64 to 0.78)
**Oklahoma**	19.68 (18.85 to 20.50)	2.06 (0.37 to 5.15)
**Oregon**	60.55 (59.19 to 61.92)	1.53 (0.86 to 2.35)
**Pennsylvania**	39.04 (38.49 to 39.60)	-0.47 (-0.81 to -0.14)
**Rhode Island**	26.91 (25.32 to 28.51)	1.81 (0.73 to 3.07)
**South Carolina**	27.62 (26.71 to 28.53)	-0.58 (-1.17 to 0.16)
**South Dakota**	26.81 (24.97 to 28.66)	2.74 (1.75 to 3.90)
**Tennessee**	25.57 (24.83 to 26.31)	-0.02 (-1.06 to 1.30)

**Texas**	20.94 (20.56 to 21.32)	-1.80 (-2.24 to -1.40)
**Utah**	24.68 (23.35 to 26.00)	-2.42 (-3.35 to -1.46)
**Vermont**	53.97 (50.84 to 57.10)	1.04 (-1.63 to 5.20)
**Virginia**	28.60 (27.88 to 29.32)	-0.48 (-1.50 to 0.68)
**Washington**	49.38 (48.39 to 50.38)	0.16 (-0.37 to 0.79)
**West Virginia**	23.85 (22.66 to 25.05)	-1.67 (-3.03 to -0.29)
**Wisconsin**	39.97 (39.07 to 40.87)	1.31 (0.93 to 1.70)
**Wyoming**	30.08 (27.32 to 32.83)	----
**U.S (Overall)**	28.62 (28.51 to 28.73)	----

Supplementary Table S6. AS associated Age-Adjusted Mortality Rates per 100,000, stratified by State in the United States, 1999 to 2020.

**Year**	**Age Adjusted Rate (95% CI)**			
	**Census Region 1: Northeast**	**Census Region 2: Midwest**	**Census Region 3: South**	**Census Region 4: West**
1999	29.84 (28.60 to 31.08)	28.53 (27.39 to 29.68)	24.17 (23.28 to 25.06)	32.02 (30.65 to 33.39)
**2000**	31.73 (30.46 to 33.00)	27.66 (26.55 to 28.78)	23.98 (23.10 to 24.86)	31.72 (30.37 to 33.07)
**2001**	30.45 (29.22 to 31.69)	27.47 (26.36 to 28.57)	23.22 (22.36 to 24.08)	30.43 (29.13 to 31.74)
**2002**	30.07 (28.85 to 31.28)	28.00 (26.89 to 29.11)	24.24 (23.36 to 25.12)	31.68 (30.36 to 32.99)
**2003**	29.44 (28.25 to 30.64)	27.43 (26.34 to 28.52)	24.66 (23.78 to 25.54)	31.85 (30.55 to 33.15)
**2004**	30.17 (28.97 to 31.38)	27.27 (26.19 to 28.35)	23.92 (23.07 to 24.78)	31.61 (30.33 to 32.89)
**2005**	29.43 (28.26 to 30.61)	29.29 (28.18 to 30.41)	23.16 (22.32 to 23.99)	34.05 (32.74 to 35.36)
**2006**	31.79 (30.58 to 33.01)	29.24 (28.14 to 30.34)	24.59 (23.74 to 25.44)	33.87 (32.59 to 35.16)
**2007**	32.71 (31.49 to 33.93)	32.80 (31.64 to 33.95)	26.40 (25.53 to 27.27)	38.15 (36.80 to 39.50)
**2008**	33.28 (32.06 to 34.50)	30.29 (29.19 to 31.39)	23.91 (23.10 to 24.73)	33.56 (32.31 to 34.80)
**2009**	33.04 (31.82 to 34.25)	30.23 (29.13 to 31.32)	23.61 (22.81 to 24.41)	34.26 (33.02 to 35.49)
**2010**	34.48 (33.25 to 35.71)	31.75 (30.63 to 32.87)	24.15 (23.35 to 24.95)	35.24 (34.00 to 36.48)
**2011**	35.11 (33.88 to 36.33)	31.75 (30.65 to 32.85)	23.55 (22.78 to 24.33)	33.45 (32.26 to 34.63)
**2012**	34.43 (33.23 to 35.63)	31.61 (30.51 to 32.70)	24.80 (24.02 to 25.59)	33.17 (32.00 to 34.33)
**2013**	35.60 (34.38 to 36.81)	32.95 (31.85 to 34.06)	24.15 (23.39 to 24.91)	34.72 (33.55 to 35.89)
**2014**	34.15 (32.97 to 35.34)	33.26 (32.16 to 34.36)	24.03 (23.28 to 24.78)	32.34 (31.22 to 33.46)
**2015**	34.61 (33.43 to 35.80)	33.69 (32.59 to 34.79)	22.74 (22.02 to 23.46)	32.37 (31.27 to 33.47)
**2016**	33.52 (32.36 to 34.68)	32.82 (31.73 to 33.90)	21.84 (21.14 to 22.53)	32.42 (31.34 to 33.51)
**2017**	31.62 (30.51 to 32.73)	31.91 (30.86 to 32.97)	22.51 (21.82 to 23.21)	30.07 (29.03 to 31.11)
**2018**	30.55 (29.47 to 31.64)	30.70 (29.68 to 31.73)	20.43 (19.77 to 21.08)	28.61 (27.62 to 29.61)
**2019**	29.63 (28.57 to 30.69)	29.63 (28.62 to 30.63)	20.13 (19.49 to 20.77)	27.94 (26.97 to 28.92)
**2020**	27.36 (26.35 to 28.38)	27.97 (27.00 to 28.94)	17.97 (17.37 to 18.57)	25.60 (24.68 to 26.53)
**Total**	32.09 (31.83 to 32.34)	30.44 (30.20 to 30.67)	23.06 (22.90 to 23.23)	32.09 (31.84 to 32.34)

Supplementary Table S7. AS associated Age-Adjusted Mortality Rates per 100,000, stratified by Census Region in the United States, 1999 to 2020

Year	Age Adjusted Rate (95% CI)			
	**Large Central Metro**	**Small Metro**	**Micropolitan (Nonmetro)**	**NonCore (Nonmetro)**
**1999**	25.19 (24.18 to 26.20)	32.09 (30.17 to 34.01)	31.94 (30.11 to 33.78)	23.63 (21.91 to 25.35)
**2000**	23.95 (22.98 to 24.93)	31.46 (29.58 to 33.34)	30.87 (29.08 to 32.66)	25.81 (24.02 to 27.59)
**2001**	24.46 (23.48 to 25.44)	30.01 (28.19 to 31.84)	28.49 (26.78 to 30.20)	26.02 (24.22 to 27.81)
**2002**	23.80 (22.84 to 24.77)	30.30 (28.48 to 32.12)	31.13 (29.34 to 32.92)	27.92 (26.06 to 29.78)
**2003**	24.23 (23.26 to 25.19)	31.70 (29.85 to 33.54)	29.95 (28.20 to 31.69)	26.29 (24.49 to 28.09)
**2004**	23.74 (22.79 to 24.69)	30.86 (29.06 to 32.66)	30.07 (28.34 to 31.81)	25.72 (23.95 to 27.50)
**2005**	23.89 (22.95 to 24.84)	33.75 (31.89 to 35.62)	30.03 (28.30 to 31.76)	26.76 (24.95 to 28.56)
**2006**	25.27 (24.31 to 26.23)	32.30 (30.50 to 34.10)	32.26 (30.49 to 34.02)	28.37 (26.52 to 30.22)
**2007**	28.25 (27.24 to 29.25)	33.36 (31.55 to 35.17)	35.44 (33.60 to 37.28)	31.90 (29.95 to 33.85)
**2008**	24.93 (23.99 to 25.86)	32.22 (30.46 to 33.97)	32.84 (31.09 to 34.60)	27.09 (25.30 to 28.88)
**2009**	24.98 (24.05 to 25.91)	33.13 (31.37 to 34.89)	32.74 (31.00 to 34.49)	29.17 (27.31 to 31.02)
**2010**	26.52 (25.58 to 27.47)	34.01 (32.24 to 35.78)	33.52 (31.77 to 35.27)	29.63 (27.77 to 31.49)
**2011**	25.71 (24.79 to 26.63)	32.13 (30.44 to 33.82)	34.01 (32.27 to 35.76)	30.49 (28.62 to 32.36)
**2012**	25.88 (24.97 to 26.79)	34.32 (32.59 to 36.05)	34.99 (33.23 to 36.75)	30.46 (28.61 to 32.31)
**2013**	26.29 (25.38 to 27.19)	34.04 (32.34 to 35.75)	35.29 (33.54 to 37.04)	31.52 (29.66 to 33.39)
**2014**	24.86 (23.98 to 25.73)	34.46 (32.76 to 36.16)	34.66 (32.93 to 36.38)	30.66 (28.82 to 32.49)
**2015**	24.52 (23.67 to 25.37)	34.27 (32.60 to 35.94)	35.53 (33.80 to 37.26)	31.64 (29.79 to 33.49)
**2016**	24.26 (23.41 to 25.10)	31.97 (30.38 to 33.57)	33.81 (32.13 to 35.49)	30.99 (29.17 to 32.81)
**2017**	22.65 (21.84 to 23.45)	32.28 (30.69 to 33.86)	32.48 (30.85 to 34.11)	28.67 (26.94 to 30.40)
**2018**	22.10 (21.31 to 22.88)	29.78 (28.27 to 31.29)	30.60 (29.03 to 32.16)	27.91 (26.22 to 29.61)
**2019**	21.23 (20.47 to 21.99)	29.56 (28.07 to 31.05)	29.56 (28.03 to 31.08)	27.63 (25.96 to 29.30)
**2020**	19.15 (18.43 to 19.87)	27.25 (25.83 to 28.66)	28.62 (27.12 to 30.11	26.08 (24.46 to 27.70)
**Total**	24.32 (24.13 to 24.51)	32.09 (31.73 to 32.46)	32.28 (31.92 to 32.65)	28.43 (28.04 to 28.81)

Supplementary Table S8. AS associated Age-Adjusted Mortality Rates per 100,000, stratified by Urban-Rural status in the United States, 1999 to 2020.
